# Predicting neighborhood-level violence from features of the physical and social environment with machine learning

**DOI:** 10.1186/s40621-025-00629-2

**Published:** 2025-11-10

**Authors:** Veronica A. Pear, Colette Smirniotis, Rose M. C. Kagawa

**Affiliations:** Centers for Violence Prevention, University of California Davis School of Medicine, 4301 X St, Sacramento, CA 95817 USA

## Abstract

**Background:**

Violence is a leading cause of death and disparity in the United States. Individuals’ physical and social environments can prevent or foster violence, but these complex milieus are challenging to model. In this study, we used machine learning to identify features of the local environment that are most predictive of violence in two Midwestern cities struggling with disinvestment and crime.

**Methods:**

This was a serial cross-sectional study of census tracts in Cleveland, Ohio and Detroit, Michigan, 2011–2019. We took a machine learning approach—extreme gradient boosting—that enabled us to model 55 neighborhood features simultaneously and without making assumptions about their relationships or functional form. These features included building quality and type, public goods and services, residential stability, socioeconomic features, historical features, and demographic features. Primary outcomes were police-reported counts per square mile of violent crime and violent crime involving a firearm in Cleveland. Secondary outcomes were homicide and firearm homicide in Cleveland and Detroit. Variable importance was assessed with Shapley values.

**Results:**

The primary models performed well, with a correlation between observed and predicted counts of 0.89 for violent crime and 0.65 for firearm-involved violent crime. For both outcomes, the variables with the highest importance tended to be in the domains of building quality and type or socioeconomic features. Several variables had high importance for both outcomes, including multifamily homes per square mile, road network density, commercial buildings per square mile, and percentage of the population that was white.

**Conclusions:**

These findings underscore the fundamental importance of place in preventing and generating violence. Future studies should explore modifiable, highly important variables as potential points of intervention.

**Supplementary Information:**

The online version contains supplementary material available at 10.1186/s40621-025-00629-2.

## Introduction

In 2020, firearm violence surpassed motor vehicle crash deaths as the leading cause of death for adolescents in the United States [1]. In 2022, there were 48,204 firearm-involved deaths, 19,651 of which were homicides [2]. In total, there were an estimated 640,710 firearm victimizations (in which an offender possessed, showed, or used a firearm during a violent crime) in 2022 [3]. Research on the social epidemiology of firearm violence prevention is nascent relative to other topics in public health, yet neighborhood-level research is critical given that efforts to alter the context in which firearm violence takes place may have greater probability of achieving broad and sustained impacts than individual-level interventions [4]. Such research requires sophisticated modeling approaches to effectively capture the dynamic and connected effects of historical, physical, economic, institutional, and social features of neighborhood environments in space and over time.

A large body of literature, much of it from the field of criminology, relates features of the physical environment to crime risk. For violent crimes, common “attractors” include schools, and particularly public secondary schools [5, 6], parks [7], bars and restaurants where alcohol is served [5, 8, 9], and public transportation sites such as bus stops [5]. Housing age and location are also potential drivers of violence. Older houses may expose children to dangerous chemicals through lead paint [10], for example. Other landscape factors such as neighborhood greenspace [11], and transportation design [12] are also associated with firearm violence and related factors such as aggression and crime.

Social and economic environments also play a critical role in neighborhood safety. Economic inequality and limited education and economic opportunities, concentrated disadvantage and poverty, and low levels of collective efficacy are all associated with community violence [13–16]. As an example, a recent study in Seattle, Washington found a strong association between neighborhood disadvantage and the count of firearm injuries at the census tract level [17].

Studies frequently attempt to isolate associations between each feature of the environment and violence individually. However, there is growing interest in using machine-learning and systems science approaches to understand socially and contextually rooted health problems such as firearm violence in more holistic ways [18–20]. For example, a previous study of predictors of firearm violence used machine learning to identify community factors associated with rates of interpersonal firearm violence within zip code tabulation areas. This study identified 18 community-level predictors, such as Black isolation and segregation indices and indicators of educational attainment and wealth, as important for predicting community rates of firearm violence [18]. Another study used deep neural networks and machine learning to identify social-contextual and individual features that were associated with acts of violence among individuals formerly involved in illegal armed groups in Colombia [20]. Studies such as these enhance our understanding of how neighborhood features work together to prevent or facilitate violence.

The current study sought to contribute to this literature by examining the importance of a wide breadth of contextual features in predicting census-tract-level violent crime in Cleveland, Ohio and Detroit, Michigan over a 9-year period. In many ways, these two cities represent the challenges stemming from deindustrialization in the United States, including long-term job loss, depopulation, and violence. Of particular note, both cities have levels of firearm violence that far exceed the national average [21]. They are also home to vibrant revitalization and community building efforts. Our findings may provide insights that are relevant not only to Cleveland and Detroit, which are worthy of study in their own right, but also to cities facing similar economic and social circumstances. Additionally, by identifying variables of particular importance in predicting neighborhood violence, we hope to inform future causal work in this area by identifying potential exposures of interest and confounders to consider in analyses.

## Methods

### Study design & sample

We conducted a serial cross-sectional study of all census tracts in Cleveland, Ohio (*n* = 177) and Detroit, Michigan (*n* = 297), 2011–2019, modeling each city separately. Neighborhoods were approximated with census tracts both because they provide small-area, stable geographic units for longitudinal analyses and because a range of variables are available at or easily converted to the census tract level. As detailed below, we paired data on a large number of census tract features with machine learning techniques to identify those features that are most predictive of violent crime.

This study was exempted by the Institutional Review Board at the University of California, Davis.

### Data & measures

We examined 55 features of the local environment hypothesized to be related to violent crime. These exposures can broadly be categorized into the following domains: building quality and type, public goods and services, residential stability, socioeconomic features, historical features, and demographic features. We also included the longitude and latitude coordinates for each census tract centroid to control for residual spatial effects [22]. All variables were measured annually, 2011–2019, unless otherwise noted and aggregated to or measured at the census-tract level. Definitions and data sources for all variables can be found in Appendix Table 1.

Variables in the domain of building quality and type included the most frequently occurring parcel type in the census tract as well as the count of each parcel type per square mile (types include single- and multi-family homes, small and large apartment buildings, condominiums, commercial buildings, industrial buildings, mixed-use parcels, and vacant lots), the percentage of parcels containing a vacant building, and the number of buildings in a census tract with outward signs of serious damage per square mile. Data are time varying, except for the damaged building measure, which were based on surveys done in 2015 in Cleveland and 2009 in Detroit.

Variables in the domain of public goods and services included the number of schools, hospitals, federally qualified health centers, religious buildings, on- and off-premises alcohol outlets, home demolitions, and home rehabilitations per square mile; the percentage of land protected from development (e.g., parks); road network density; the minimum distance to public transportation; and the National Walkability Index score. The earliest year of data available for schools was 2015; these data were therefore carried backward for 2011–2014. Data for alcohol outlets were available in 2010–2015 and 2017; values for 2016 were interpolated and the values from 2017 was carried forward. The percentage of land protected from development and road network density were measured in 2018 and values were carried forward and backward across the study period. Distance to a transit stop and National Walkability Index values were measured in 2020 and carried backwards. The remaining variables were measured annually.

Residential stability variables included the percentage of occupied housing units that were renter-occupied, recent movers, the percentage of parcels sold or transferred to a new owner in the past 5 years, and the maximum number of sales or transfers for a single parcel.

Socioeconomic features included census tract measures of employment (percentage unemployed and percentage out of the labor force), income (percentage working in high-paying professions, median household income, and percentage below 150% of the poverty line), education (percentage with at least a high school education, percentage of children enrolled in preschool, and percentage of children in private school), female-headed households, individuals serving in the armed forces, residential economic segregation, households with crowding, population using public transportation, median residential sales price, and parcels that are tax delinquent or foreclosed. Residential economic segregation was measured with the index of concentration at the extremes for income [23]. Overcrowding was defined following the Department of Housing and Urban Development and others as more than 1.5 persons per room (where rooms include all living areas) [24].

Historical features included the Home Owners’ Loan Corporation redlining grade. These grades were converted to numeric values (ranging from 1 for “A” [green] to 4 for “D” [red]). The tract-level data was created by calculating weighted averages of these values based on the proportion of a tract’s area that was assigned a given grade [25].

Demographic features included census tract population count, percentage of population that are young men aged 15 to 29, racial and ethnic distributions (Hispanic or Latino of any race, Black alone, white alone, other or multi-race), racial residential segregation, and naturalized and non-citizen immigrants. Black-white racial segregation was measured with the index of concentration at the extremes for race [23].

The outcomes of interest were rates of violence and firearm violence, though how these were measured differed between the two cities due to variation in data availability. In Cleveland, the outcomes were measured as annual counts of Part 1 violent crime (homicide, rape, robbery, and aggravated assault), Part 1 violent crime involving a firearm (homicide, robbery, and aggravated assault with a firearm), homicide, and firearm homicide using data provided by the Cleveland Police Department. In all cases, the census tract area in square miles was included as an offset so the outcome could be interpreted as a the rate per square mile. The coding of firearm crimes changed in Cleveland during the study period; models for firearm-specific outcomes are therefore limited to 2016–2019, after the change occurred.

Crime data with sufficiently accurate location indicators were not available for Detroit. Instead, we examined homicide and firearm homicide using death records provided by the Michigan Department of Health and Human Services. In these records, location is based on decedent residence rather than the location of the incident. Due to the low predictive accuracy of models using homicide data in both Cleveland and Detroit (Appendix Table 2), these are explored as secondary outcomes. As a result, our primary analyses focus more heavily on Cleveland.

### Statistical analysis

To assess the importance of census tract-level exposures in predicting violence, we calculated Shapley values, which provide the average marginal contribution of each independent variable to the predicted outcome [26]. In brief, Shapley values are calculated for each variable by first predicting the outcome for all possible combinations of covariates, and for each combination, the contribution of the variable to the prediction is quantified. These contributions are then averaged to provide a summary importance measure for the variable. As this can get computationally intensive very quickly, a sample of the observed covariate combinations is used in practice.

The underlying predictions used in the Shapley calculations were based on machine learning in the form of gradient boosted regression trees (XGBoost), which enabled us to build an accurate predictive model without assumptions about the underlying data distribution. These types of models are particularly well suited for data involving multicollinearity, non-independent observations, and missingness [27]. XGBoost is an iterative ensemble model in which a series of models are trained one after another in succession, with each new model being trained to correct the errors (residuals) of the previous. We followed Sheetal et al. in applying XGBoost to longitudinal data, predicting violence in each year based on variables in that year and all prior years [27]. Hyperparameters were tuned using Model-Based Optimization to minimize the negative log-likelihood. We held a 20% random sample for testing and trained the model on the other 80%. Models were run separately for Cleveland and Detroit and for each outcome of interest, with census tract area included as an offset. Model performance was measured comparing the predicted and observed values of the outcome (annual census tract counts of violence) using a correlation coefficient and the root mean squared error.

### Sensitivity analyses

Having many highly correlated variables in a model may result in smaller Shapley values for those variables, since the quantified importance of the underlying construct is divided between the associated variables. To explore this, we conducted sensitivity analyses that removed highly correlated variables. We first identified variables that were highly correlated (*r*>|0.6|) within each a priori exposure category of interest. We then removed one variable from each highly correlated pair in a way that minimized the total number of variables being removed (e.g., if variables A and B were highly correlated and A was also highly correlated with C, we would remove A to break both correlations parsimoniously).

## Results

Table 1 displays descriptive statistics for the exposure and outcome variables pooled across Cleveland’s 177 census tracts over the study period. The most common parcel type is single-family home, comprising a plurality in 66% of census-tract-years. Census tracts had a median of 1.6 on-premises alcohol outlets and 0.0 off-premises alcohol outlets per square mile. The median percentage of occupied housing units that were renter-occupied over the study period was nearly 60%. The median census-tract-year percentage of population below 150% of the poverty line was 41.3%. Demographically, the median census-tract-year percentage of residents that were Black alone was 68.7%. The median number of Part 1 violent crimes was 27 and the median number of Part 1 firearm violence crimes was 4.

**Table 1:** Characteristics of Cleveland Census Tracts, 2011–2019

Characteristic^a^	Census tract years (*N* = 1,593)^b^
***Building quality & type***	
Damaged buildings/sq mi	60.2 (14.8, 183.4)
NA	9
Plurality of parcel types	
Commercial buildings	90 (5.6%)
Condominiums	63 (4.0%)
Industrial buildings	57 (3.6%)
Large multi-unit apartments	18 (1.1%)
Multi-family homes	113 (7.1%)
Other	195 (12.2%)
Single family homes	1,049 (65.9%)
Vacant lots	8 (0.5%)
Single family homes/sq mi	1,158.1 (697.8, 1,808.7)
Small apartment buildings/sq mi	26.5 (6.7, 56.5)
Multifamily homes/sq mi	473.2 (163.4, 830.6)
Large apartment buildings/sq mi	7.9 (2.7, 21.2)
Condominiums/sq mi	0.0 (0.0, 0.0)
Commercial buildings/sq mi	85.8 (52.3, 126.7)
Industrial buildings/sq mi	16.0 (0.0, 51.7)
Vacant lots/sq mi	146.8 (72.1, 260.9)
Mixed-use parcels/sq mi	24.3 (9.5, 47.7)
Parcels with vacant buildings, %	20.6 (12.2, 30.5)
NA	9
***Public goods & services***	
Primary and secondary education schools/sq mi	1.5 (0.0, 4.0)
Hospitals/sq mi	0.0 (0.0, 0.0)
Federally qualified health centers/sq mi	0.0 (0.0, 0.0)
Land protected from development, %	0.7 (0.0, 6.9)
Religious buildings/sq mi	5.1 (2.3, 10.8)
On-premises alcohol outlets/sq mi	1.6 (0.0, 5.0)
Off-premises alcohol outlets/sq mi	0.0 (0.0, 0.0)
Road network density	22.4 (19.1, 25.3)
Minimum distance to nearest transit stop	2.0 (1.5, 2.5)
National Walkability Index	13.1 (12.0, 14.3)
Demolitions/sq mi	10.7 (1.7, 31.8)
Rehabilitations/sq mi	0.0 (0.0, 4.0)
***Residential stability***	
Occupied housing units renter-occupied, %	59.2 (47.4, 68.2)
NA	19
Recent movers^c^	102.0 (79.0, 131.0)
Parcels with arm’s length transfer in past 5 years, %	12.1 (9.2, 14.7)
NA	28
Maximum number of arm’s length transfers for a single parcel	
1	17 (1.1%)
2	215 (13.5%)
3	504 (31.6%)
4	490 (30.8%)
5	231 (14.5%)
6	71 (4.5%)
7	30 (1.9%)
8	8 (0.5%)
NA	27
***Socioeconomic features***	
Population aged 16 + unemployed, %	7.1 (4.6, 10.0)
NA	19
Population aged 16 + out of the labor force, %	42.7 (36.2, 48.8)
NA	19
Employed civilian population 16 + working in high-paying professions, %	12.6 (8.9, 16.5)
NA	20
Population aged 25 + with at least a high school education, %	67.4 (59.5, 74.6)
NA	20
Kids aged 3 + enrolled in nursery/preschool, %	2.3 (1.5, 3.1)
NA	19
Kids enrolled in K-12 in private school, %	12.2 (5.8, 23.1)
NA	21
Female-headed households, %	16.1 (10.5, 19.2)
NA	19
Currently in the armed forces^c^	0.0 (0.0, 0.0)
Population below 150% of poverty line, %	41.3 (22.9, 55.9)
NA	19
Residential economic segregation	−0.5 (−0.6, −0.3)
NA	19
Median household income	27,013 (20,771, 34,534)
Households with crowding	3.0 (0.0, 7.0)
Population using public transportation^c^	267.0 (147.0, 405.0)
Median residential sales price in past 5 years	20,000 (12,929.5, 40,500)
NA	50
Parcels tax delinquent, %	16.0 (7.7, 24.2)
Parcels tax foreclosed, %	0.8 (0.3, 1.5)
Parcels mortgage foreclosed, %	1.1 (0.6, 1.8)
***Historical features***	
HOLC’s redlining grade	3.3 (3.0, 4.0)
NA	54
***Demographic features***	
Population aged 15 to 29 and male, %	10.8 (9.2, 12.0)
NA	19
Population	2,115 (1,468, 2,859)
Racial residential segregation	−0.5 (−1.0, 0.3)
NA	19
Population Hispanic or Latino, %	3.6 (1.4, 15.5)
NA	19
Population Black alone, %	68.7 (20.5, 99.0)
NA	19
Population white alone, %	24.0 (0.2, 56.9)
NA	19
Population other race or 2 + races, %	3.4 (0.6, 12.5)
NA	19
Population foreign born and naturalized^c^	33.0 (7.0, 74.0)
Population foreign born and not a citizen^c^	26.0 (6.0, 53.0)
***Crime and violence***	
UCR Part 1 Violent Crimes, N	27.0 (17.0, 38.0)
UCR Part 1 Firearm Violence Crimes (2016–2019), N	4.0 (2.0, 8.0)

^a^Full variable definitions and data sources can be found in Appendix Table 1. "NA" indicates the number of census tract years for which the value does not apply (the relevant denominator is equal to zero)

^b^Continuous variables: Median (Q1, Q3). Categorical variables: n (%)

^c^Variable has a range is 0-1000 and is scaled such that 100 is the national average

The Cleveland XGBoost model performance metrics are displayed in Table 2. The correlation coefficient for predicted and observed values indicated good model performance for both Part 1 violence (*r* = 0.89) and, to a lesser degree, Part 1 firearm violence (*r* = 0.65). See Appendix Table 3 for details on the hyperparameter space explored and the final hyperparameter values modeled. Model performance using the fatal outcomes (homicide and firearm homicide) was poor in both Cleveland and Detroit (Appendix Table 2).

**Table 2:** XGBoost Model Performance, Cleveland*

Outcome	Observed Mean (SD)	Predicted Mean (SD)	Pearson’s *r*	RMSE
Pt 1. Violence	29.27 (18.22)	29.71 (15.84)	0.89	8.40
Pt. 1 Firearm Violence	5.44 (4.43)	5.60 (3.80)	0.65	3.50

* Measured using the testing data

**Fig. 1:**
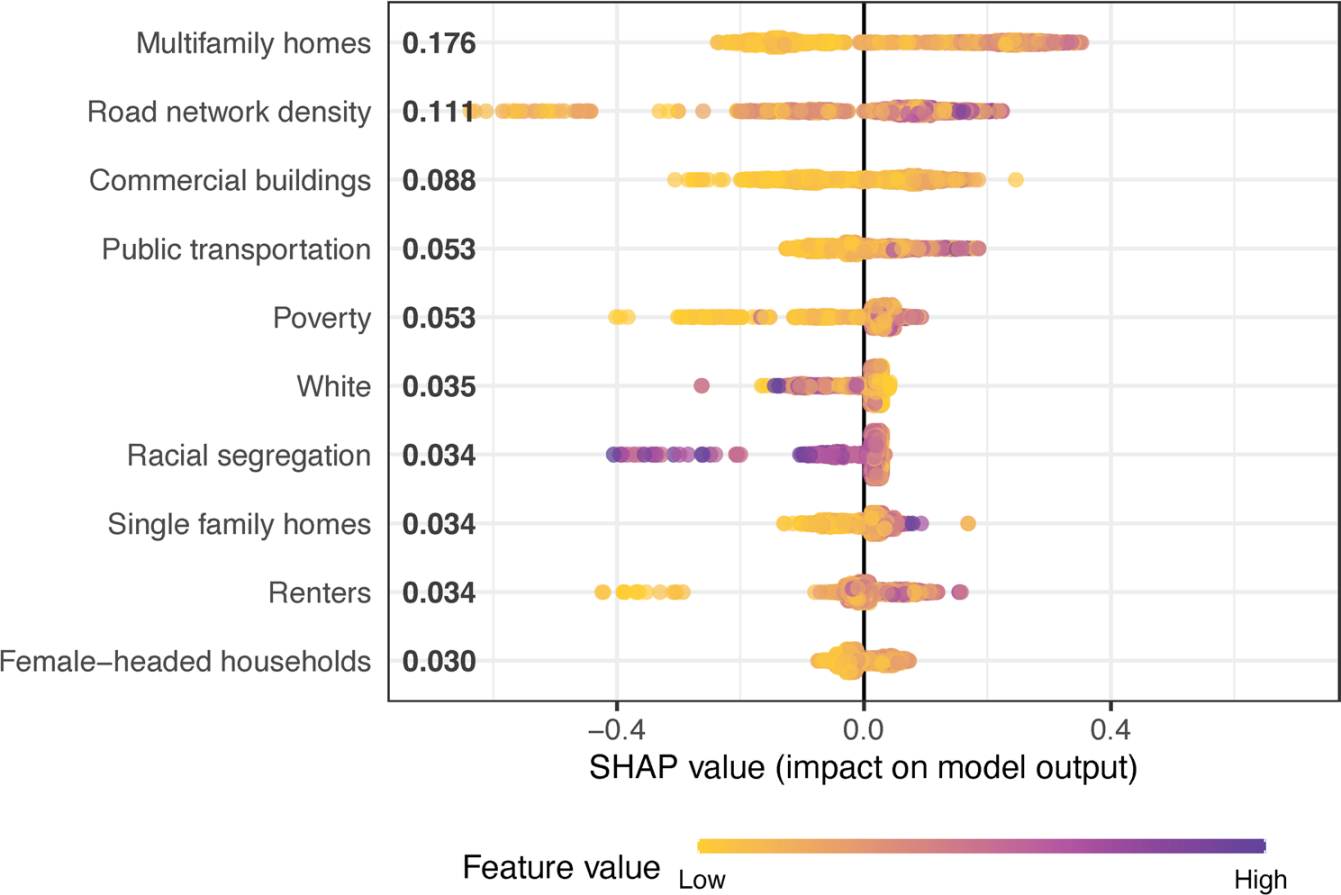
Top 10 Predictors of Pt. 1 Violence in Cleveland, Shapley Value by Feature Value

**Fig. 2:**
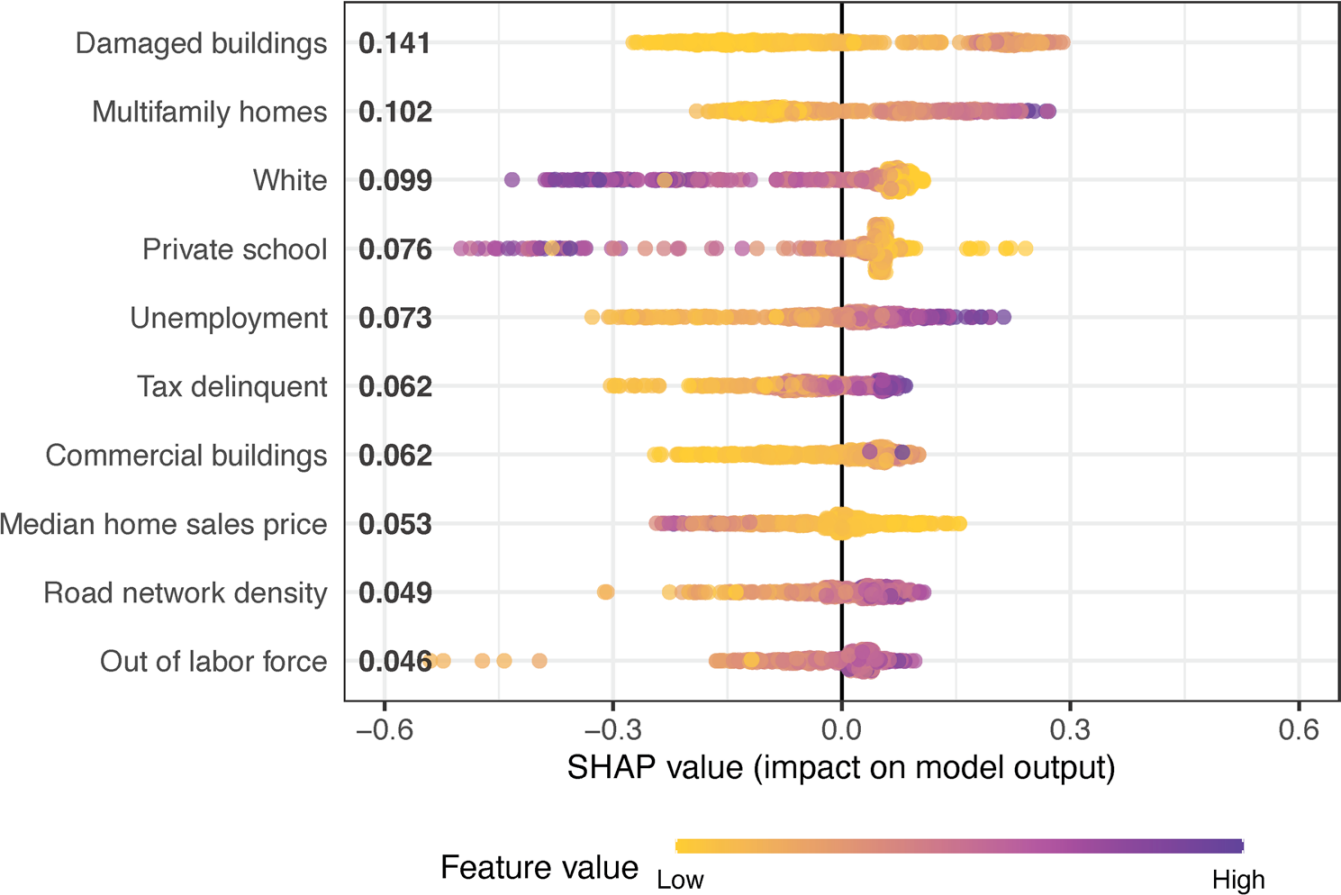
Top 10 Predictors of Pt. 1 Firearm Violence in Cleveland, Shapley Value by Feature Value

The variables with the highest 10 Shapley values are displayed in Table 3 and graphically in Fig. 1 (Part 1 violence) and Fig. 2 (Part 1 firearm violence). For both outcomes in Cleveland, the most important variables tended to be in the domains of building quality and type or socioeconomic features. Both outcomes had several variables in common across their 10 top lists: multifamily homes per square mile, road network density, commercial buildings per square mile, and percentage white alone. Higher values of multifamily homes, road network density, and commercial buildings were associated with higher values of the outcomes, where the inverse relationship held for percentage white.

**Table 3:** Ten Most Important Predictors of Violence in Cleveland

Outcome	Variable	Category	Mean Shapley Value
Pt. 1 Violence*	Multifamily (2–4 family) homes per square mile	Building quality & type	0.1761
	Road network density	Public goods & services	0.1113
	Commercial buildings per square mile	Building quality & type	0.0880
	Percent of population that uses public transportation	Socioeconomic features	0.0533
	Percent below 150% of the poverty line	Socioeconomic features	0.0526
	Percent white	Demographic features	0.0347
	Racial residential segregation	Demographic features	0.0339
	Single family homes per square mile	Building quality & type	0.0336
	Percent housing units occupied by renters	Residential stability	0.0336
	Percent female-headed family households	Socioeconomic features	0.0297
Pt 1. Firearm Violence	Buildings per square mile graded D or F on quality	Building quality & type	0.1414
	Multifamily (2–4 family) homes per square mile	Building quality & type	0.1025
	Percent white	Demographic features	0.0994
	Percent children enrolled in K-12 that are in private school	Socioeconomic features	0.0759
	Percent unemployed	Socioeconomic features	0.0726
	Percent of parcels that are tax delinquent	Socioeconomic features	0.0624
	Commercial buildings per square mile	Building quality & type	0.0622
	Median residential sales price in previous 5 years	Socioeconomic features	0.0527
	Road network density	Public goods & services	0.0494
	Percent out of labor force	Socioeconomic features	0.0462

* X and Y coordinates were both in the top 10 Shapley values for Part 1 violence but are not included in the table as they were included only to control for spatial autocorrelation

Many of the remaining variables with the top 10 highest Shapley values in Cleveland were markers of income and socioeconomic status, though the exact variables that rose to the top varied between the two models. For Part 1 violence, these variables included population using public transportation, population below 150% of the poverty line, racial residential segregation, single family homes per square mile, percentage of occupied housing units that are renter occupied, and percentage of female-headed households. For Part 1 firearm violence, the remaining variables included damaged buildings per square mile, percentage of children enrolled in private school, percentage unemployed, percentage of parcels that are tax delinquent, median home sales price, and percentage out of the labor force.

Results for the secondary outcomes of homicide and firearm homicide for both Cleveland and Detroit are displayed in Appendix Table 4. The percentages of female-headed households and Black residents had high variable importance in all four models. Single family homes, large apartment buildings, and damaged buildings per square mile each had high importance in 3 of the 4 models. The percentage of parcels that were tax delinquent was important only in the firearm homicide models. Other important variables seemed to cluster by city; residential racial segregation, small apartment buildings per square mile, and populationroad network density appeared as highly important variables in both the firearm homicide and homicide models for Detroit, while unemployment was highly important for both models in Cleveland. 

We removed 18 highly correlated variables in the sensitivity analysis (Appendix Table 5). The model performance was essentially unchanged from the primary analysis (Appendix Table 6). Variables with the highest Shapley values were largely similar to the results from the primary analysis (Appendix Table 7). Variables that no longer appeared in the list of top-10 Shapley values were almost always those that were removed due to collinearity. For Part 1 firearm violence, these were replaced with variables capturing the geographic consequences of systemic racism, including racial residential segregation, economic residential segregation, and historic redlining grade.

## Discussion

In this study, we quantified variable importance with machine learning to identify contextual features of the local environment that are most predictive of violence. We examined 55 features of census tracts in Cleveland and Detroit, two Midwestern cities that have struggled with deindustrialization, disinvestment, and violence. Using only these local characteristics, we were able to predict Part 1 violent crimes and Part 1 firearm violence in Cleveland with a high degree of accuracy. In conjunction with the wider literature, these findings highlight the fundamental role of place to the etiology of violence. Features identified as most important can be considered in future research modeling these outcomes and, if modifiable, explored in causal analyses that could identify potential preventive interventions.

Census tract features that described mixed-use areas of the city, with greater densities of multi-family homes, renters, roads, and commercial buildings and where a greater percentage of the population used public transportation, were among the most predictive of elevated rates of violent crime. Such areas are home to many known criminogenic features, including a more transient population (which can limit social cohesion in an area [28]), transportation stops [29], and commercial buildings [30, 31]. Social disorganization theory is useful for interpreting these results [32], as urban areas with greater traffic and more strangers are thought to erode the ability of residents to maintain informal social controls. Routine activities theory, which suggests that potential offenders and targets cross paths as people engage in daily activities, such as going to school, work, and the grocery store [33], is also consistent with our results, as areas marked by greater commercial activity and populations in transit bring together potential targets and offenders with greater frequency, increasing opportunities for crime.

These findings are generally consistent with studies in other American cities. For example, a study using data from Los Angeles found a strong association between mixed land use levels and violent crime rates in areas of greater social and economic advantage [31]. However, a study in Columbus, Ohio found that areas with greater commercial and residential density had elevated rates of robbery but lower rates of aggravated assault and homicide; the authors hypothesized that assault and homicide were more responsive to the effects of informal social controls present in mixed-used areas [30]. Differences between studies could be explained by the differing study populations, time periods, and methods used.

Not surprisingly, socioeconomic status also appears to play an important role in crime prediction. Elevated rates of poverty, and greater proportions of renters and female-headed households were predictive of higher Part 1 violent crime rates, while higher unemployment and tax delinquency rates as well as a greater proportion of the population being out of the labor force were all predictive of higher rates of firearm violence. These findings are in line with a vast literature documenting the criminogenic effects of entrenched poverty and economic disinvestment. The pathways linking poverty and its associated contexts to crime are numerous and complex [34]. Unemployment may push people to engage in the illicit labor market where criminal activities may constitute or supplement income that is otherwise hard to come by [35]. At the same time, employment and the social connections it brings, is one of the many pro-social bonds that connects people to institutions and society. Lower levels of employment, and perhaps particularly, higher rates of having left the labor force completely, serve as direct motivators for criminal engagement, indicators of weak social ties, and signs of reduced economic opportunity. Elevated rates of family disruption, as proxied by female-headed households, could be related to joblessness, incarceration, or other factors [36], and higher proportions of renters are signs of a more transient population that may have weaker informal social controls.

What is surprising is that many of the socioeconomic measures we analyzed were ranked lower in importance than the land use features. While the built environment features and the socioeconomic features are likely linked through (often discriminatory) land use and lending laws, the prominence of the built environment features may indicate they are better proxies for low collective efficacy. While socioeconomic disadvantage is associated with violence, it is moderated by collective efficacy, which serves as a protective factor against violence [15]. Our results could be explained by lower levels of collective efficacy in higher density, commercial areas than in areas marked by social and economic disadvantage.

The strongest predictors for violent crime involving firearms overlapped in several cases with the strongest predictors for violent crime more generally. However, they differed in a few notable ways. Standard measures of socioeconomic disadvantage, such as the poverty rate and the percentage of households that are female-headed, showed up as leading predictors for violent crime but not for violent firearm-involved crime. The features most predictive of firearm violence (and not general violence) suggested a more acute level of neighborhood distress. These included employment measures (unemployment and out of labor force), tax delinquency, sales values, and home quality. These are characteristics of a severely unhealthy housing market, where home values are worth less than the mortgage. Coupled with high levels of unemployment and the proliferation of homes in poor condition, these features are indicative of neighborhoods with immense structural barriers to financial wellbeing.

In the sensitivity analysis removing highly correlated variables, several predictors related to systemic racism and its consequences came to the fore, especially in the firearm violence analysis. These include racial residential segregation, HOLC redlining grade, and residential economic segregation. These measures indicate long-term, racialized, place-based disinvestment and have been found to be associated with firearm violence previously [30, 37]. The fact that they emerged as top-ranking predictors only after correlated variables were removed may suggest that some of the variables removed were acting as mediators (reducing the importance of these more distal variables when both were included in the model together). This could be explored in future analyses.

### Limitations

This study’s findings should be interpreted in light of its limitations. In secondary analyses examining homicide and firearm homicide, the XGBoost models had poor predictive power, likely due to the rarity of the outcomes. Because they performed poorly, results from these analyses should be interpreted with caution and taken merely as suggestive. Furthermore, there is not a standard approach to controlling for spatial autocorrelation in XGBoost models, and our approach of including census tract centroid coordinates was imperfect; there was likely residual autocorrelation as a result. In addition, this study examined two large cities in the Midwest and results may not generalize to other populations. Relative to other similarly sized cities, Detroit and Cleveland have high poverty rates; it is possible that the features we identified here would function differently in areas with lower poverty and greater resources. We were unable to explore interactions between census tract factors and poverty because they were highly correlated; as a result, we were unable to disentangle the unique roles of each. Future research could explore this empirically by replicating this study in geographically and culturally distinct cities across the US. Finally, we examined associations for census tracts, which do not necessarily align with meaningful neighborhoods within cities; findings may have been different if we had used a different unit of analysis.

## Conclusions

Findings from this study support the hypothesis that the local environment plays an important role in violence, as we were able to accurately predict violent crime using only these features. This suggests that neighborhood-level interventions should be explored for prevention. Of particular interest is the role of land use and housing type. Our hypothesis-generating study provides new directions for future causal work investigating such interventions and can also inform researchers interested in studying and predicting violent crime in urban areas.

## Supplementary Information

Supplementary Material 1

## Data Availability

The datasets used and/or analyzed during the current study are available from the corresponding author on reasonable request.
