# Supplementary material for: Predicting neighborhood-level violence from features of the physical and social environment with machine learning
Source: Inj Epidemiol. 2025 Nov 10;12:75. doi: 10.1186/s40621-025-00629-2 (PMC12604338; doi:10.1186/s40621-025-00629-2)
Supplement: Supplementary file 1 — Supplementary Material 1 [file 40621_2025_629_MOESM1_ESM.docx]

**Predicting Neighborhood-Level Violence from Features of the Physical and Social Environment with Machine Learning: APPENDIX**

Pear VA, Smirniotis C, Kagawa RMC

*Injury Epidemiology*

**Appendix Table 1. Exposure and Outcome Variable Details**

| **Variable** | **Definition*** | **Source** |
| --- | --- | --- |
| ***Building Quality & Type*** |  |  |
| Damaged buildings/sq mi | Count of buildings per square mile with a quality grade of D or F in Cleveland (2015) or labeled “poor” or “demolished” in Detroit (2009) | Cleveland: Cleveland Property Survey  Detroit: Detroit Residential Parcel Survey |
| Plurality of parcel types | Census tracts are assigned the parcel type that constitutes a plurality of parcels within its limits each year | Cleveland: Cuyahoga County Fiscal Office  Detroit: Detroit Office of the Assessor |
| Single family homes/sq mi | Annual count of single-family homes per square mile | Cleveland: Cuyahoga County Fiscal Office  Detroit: Detroit Office of the Assessor |
| Small apartment buildings/sq mi | Annual count of small (1-6 unit) apartment buildings per square mile | Cleveland: Cuyahoga County Fiscal Office  Detroit: Detroit Office of the Assessor |
| Multi-family homes/sq mi | Annual count of multi-family (2-4 family) homes per square mile | Cleveland: Cuyahoga County Fiscal Office  Detroit: Detroit Office of the Assessor |
| Large apartment buildings/sq mi | Annual count of large (7+ unit) apartment buildings per square mile | Cleveland: Cuyahoga County Fiscal Office  Detroit: Detroit Office of the Assessor |
| Condominiums/sq mi | Annual count of condominiums per square mile | Cleveland: Cuyahoga County Fiscal Office  Detroit: Detroit Office of the Assessor |
| Commercial buildings/sq mi | Annual count of commercial buildings per square mile | Cleveland: Cuyahoga County Fiscal Office  Detroit: Detroit Office of the Assessor |
| Industrial buildings/sq mi | Annual count of industrial buildings per square mile | Cleveland: Cuyahoga County Fiscal Office  Detroit: Detroit Office of the Assessor |
| Vacant lots/sq mi | Annual count of vacant lots per square mile | Cleveland: Cuyahoga County Fiscal Office  Detroit: Detroit Office of the Assessor |
| Mixed-use parcels/sq mi | Annual count of parcels with both livable and non-livable space (such as retail or offices) per square mile | Cleveland: Cuyahoga County Fiscal Office  Detroit: Detroit Office of the Assessor |
| Parcels with vacant buildings, % | Annual percent of parcels in a census tract that are vacant | Geolytics |
| ***Public Goods & Services*** |  |  |
| Primary and secondary education schools/sq mi | Annual count of public primary and secondary public schools per square mile (data start in 2015) | National Center for Education Statistics |
| Hospitals/sq mi | Annual count of hospitals per square mile | Centers for Medicare and Medicaid Services |
| Federally qualified health centers/sq mi | Annual count of federally qualified health centers per square mile | Centers for Medicare and Medicaid Services |
| Land protected from development, % | Percent of acres in a census tract protected from development (e.g., parks, natural areas, or conservation areas) in 2018 | Environmental Protection Agency’s Smart Location Database |
| Religious buildings/sq mi | Number of buildings designated as places of worship per square mile, based on records from 2007-2009 | Homeland Infrastructure Foundation-Level Data (HIFLD) Open – Places of Worship |
| On-premises alcohol outlets/sq mi | Annual count of on-premises drinking establishments per square mile (e.g., bars and restaurants) | National Neighborhood Data Archive: Eating and Drinking Places by Census Tract, United States, 2003-2017 |
| Off-premises alcohol outlets/sq mi | Annual count of establishments selling alcohol for off-premises consumption per square mile (e.g., liquor stores) | National Neighborhood Data Archive: Liquor, Tobacco, and Convenience Stores by Census Tract, United States, 2003-2017 |
| Road network density | Total road network miles per square mile in 2018 | Environmental Protection Agency’s Smart Location Database |
| Minimum distance to nearest transit stop | Census tract mean of component block group (city-specific) tertiles for the minimum walking distance in meters from the population-weighted centroid to the nearest transit stop in 2020; 1 is close, 2 is mid-distance, and 3 is far. | Environmental Protection Agency’s Smart Location Database |
| National Walkability Index | Census tract mean of component black group National Walkability Index scores in 2020; ranges from 1 (least walkable) to 20 (most walkable). | Environmental Protection Agency’s Smart Location Database |
| Demolitions/sq mi | Annual count of demolished buildings per square mile | Cleveland: Cuyahoga Land Bank  Detroit: Detroit Land Bank Authority |
| Rehabilitations/sq mi | Annual count of rehabilitated buildings per square mile | Cleveland: Cuyahoga Land Bank  Detroit: Detroit Land Bank Authority |
| ***Residential Stability*** | | |
| Occupied housing units renter-occupied, % | Annual percent of occupied housing units that are renter occupied | Geolytics |
| Recent movers | Annual measure of recent movers; scale is 0-1000 and national mean is 100. | Geolytics |
| Parcels with arm’s length transfer in past 5 years, % | Annual percent of parcels undergoing arm’s length transfers in the previous 5 years | Cleveland: Cuyahoga County Fiscal Office  Detroit: Wayne County Register of Deeds |
| Maximum number of arm’s length transfers for a single parcel in past 5 years | Annual maximum count of arm’s length transfers for a single parcel in the previous 5 years | Cleveland: Cuyahoga County Fiscal Office  Detroit: Wayne County Register of Deeds |
| ***Socioeconomic Features*** | | |
| Population aged 16+ unemployed, % | Annual percent of population aged 16+ who are unemployed | Geolytics |
| Population aged 16+ out of the labor force, % | Annual percent of population aged 16+ who are out of the labor force | Geolytics |
| Employed civilian population 16+ working in high-paying professions, % | Annual percent of employed civilian population aged 16+ working in professional, business, or financial sectors | Geolytics |
| Population aged 25+ with at least a high school education, % | Annual percent of population aged 25+ who have at least a high school education | Geolytics |
| Kids aged 3+ enrolled in nursery/preschool, % | Annual percent of children aged 3+ enrolled in nursery or preschool | Geolytics |
| Kids enrolled in K-12 in private school, % | Annual percent of children enrolled in K-12 education that are in private school | Geolytics |
| Female-headed households, % | Annual percent of households headed by unmarried women with children under 18 | Geolytics |
| Currently in the armed forces | Annual measure of population currently in the armed forces; scale is 0 to 1000 and national mean is 100. | Geolytics |
| Population below 150% of poverty line, % | Annual percent of population that is below 150% of the poverty line | Geolytics |
| Residential economic segregation | Annual index of concentration at the extremes for income:  ICE_i_ = (A_i_ − P_i_)/T_i_  Where, for a given census tract, A_i_ is the number of households making >=$100,000, P_i_ is the number of households making <$25,000, and T_i_ is the total count of households with income data. Values range from -1 (entirely disadvantaged) to 1 (entirely advantaged). | Source data from Geolytics |
| Median household income | Annual median household income in dollars | Geolytics |
| Households with crowding, N | Annual count of households with more than 1.5 persons per room | Geolytics |
| Population using public transportation | Annual measure of population using public transportation; scale is 0-1000 and national mean is 100. | Geolytics |
| Median residential sales price in past 5 years | Annual median residential sales price in previous 5 years | Cleveland: Cuyahoga County Fiscal Office  Detroit: Wayne County Register of Deeds |
| Parcels tax delinquent, % | Annual percent of parcels that are tax delinquent (owe $1 or more) | Cleveland: Cuyahoga County Fiscal Office  Detroit: Wayne County Treasurer |
| Parcels tax foreclosed, % | Annual percent of parcels that are tax foreclosed | Cleveland: Cuyahoga County Common Pleas Court  Detroit: Wayne County Treasurer |
| Parcels mortgage foreclosed, % | Annual percent of parcels with buildings that are mortgage foreclosed | Cleveland: Cuyahoga County Common Pleas Court  Detroit: Wayne County Treasurer |
| ***Historical Features*** | | |
| HOLC’s redlining grade | Home Owners' Loan Corporation (HOLC)'s historical redlining grade (1=green, 4=red) | Historic Redlining Scores for 2010 and 2020 US Census Tracts |
| ***Demographic Features*** | | |
| Population aged 15 to 29 and male, % | Annual percent of population aged 15-29 and male | Geolytics |
| Population | Annual population count | Geolytics |
| Racial residential segregation | Annual index of concentration at the extremes for race:  ICE_i_ = (A_i_ − P_i_)/T_i_  Where, for a given census tract, A_i_ is the number of non-Hispanic white residents, P_i_ is the number of Black residents, and T_i_ is the total count of people race/ethnicity data. Values range from -1 (entirely Black) to 1 (entirely non-Hispanic white). | Source data from Geolytics |
| Population Hispanic or Latino, % | Annual percent of the population that is Hispanic or Latino (any race) | Geolytics |
| Population Black alone, % | Annual percent of the population that is non-Hispanic Black alone | Geolytics |
| Population white alone, % | Annual percent of the population that is non-Hispanic white alone | Geolytics |
| Population other race or 2+ races, % | Annual percent of the population that is non-Hispanic Native American, Asian, Pacific Islander, or 2+ races | Geolytics |
| Population foreign born and naturalized | Annual measure of population that is foreign born and naturalized; scale is 1-1000 and national mean is 100 | Geolytics |
| Population foreign born and not a citizen | Annual measure of population that is foreign born and not a citizen; scale is 1-1000 and national mean is 100 | Geolytics |
| ***Crime and Violence*** | | |
| UCR Part 1 Violent Crimes, N | Annual count of part 1 violent crimes: homicide, rape, robbery, and aggravated assault reported to police | Cleveland: Cleveland Police Department |
| UCR Part 1 Firearm Violence Crimes, N | Annual count of homicide, robbery, and aggravated assault with a firearm reported to police, 2016-2019 | Cleveland: Cleveland Police Department |
| Homicide | Annual count of homicides reported to police | Cleveland: Cleveland Police Department  Detroit: Michigan Department of Health and Human Services |
| Firearm Homicide | Annual count of homicides involving a firearm reported to police | Cleveland: Cleveland Police Department  Detroit: Michigan Department of Health and Human Services |

* All variables are aggregated to or measured at the census tract level.

**Appendix Table 2. XGBoost Model Performance,* Secondary Outcomes**

| **Outcome** | **Observed**  Mean (SD) | **Predicted**  Mean (SD) | **Pearson’s *r*** | **RMSE** |
| --- | --- | --- | --- | --- |
| **CLEVELAND** |  |  |  |  |
| Firearm  Homicide | 0.49 (0.86) | 0.39 (0.31) | 0.39 | 0.80 |
| Homicide | 0.46 (0.75) | 0.50 (0.40) | 0.32 | 0.73 |
| **DETROIT** |  |  |  |  |
| Firearm  Homicide | 0.57 (0.90) | 0.60 (0.33) | 0.44 | 0.81 |
| Homicide | 0.81 (1.02) | 0.81 (0.44) | 0.38 | 0.94 |

* Measured using the testing data

**Appendix Table 3. XGBoost Hyperparameter Space Explored and Final Values Used**

| **Parameter** | **Type** | **Minimum** | **Maximum** | **Final Used** |
| --- | --- | --- | --- | --- |
| **CLEVELAND** |  |  |  |  |
| **Pt. 1 Violence** |  |  |  |  |
| eta | Numeric | 0.001 | 0.5 | 0.0109 |
| gamma | Numeric | 0 | 4 | 2.17 |
| max_depth | Integer | 1 | 15 | 4 |
| min_child_weight | Integer | 0 | 5 | 1 |
| subsample | Numeric | 0.25 | 1.0 | 0.619 |
| colsample_bytree | Numeric | 0.5 | 0.99 | 0.958 |
| nrounds | Integer | 1 | 7000 | 2335 |
| **Pt. 1 Firearm Violence** |  |  |  |  |
| eta | Numeric | 0.001 | 0.5 | 0.068 |
| gamma | Numeric | 0 | 4 | 3.240 |
| max_depth | Integer | 1 | 15 | 6 |
| min_child_weight | Integer | 0 | 5 | 2 |
| subsample | Numeric | 0.25 | 1.0 | 0.270 |
| colsample_bytree | Numeric | 0.5 | 0.99 | 0.969 |
| nrounds | Integer | 1 | 7000 | 287 |

**Appendix Table 4. Ten Most Important Predictors of Violence, Secondary Outcomes**

| **Outcome** | **Variable** | **Category** | **Mean Value** |
| --- | --- | --- | --- |
| **CLEVELAND** |  |  |  |
| Firearm Homicide | Residential economic segregation | Socioeconomic features | 0.1232 |
|  | Population aged 16+ unemployed, % | Socioeconomic features | 0.1230 |
|  | Damaged buildings/sq mi, N | Building quality & type | 0.1085 |
|  | Large apartment buildings/sq mi, N | Building quality & type | 0.0854 |
|  | Female-headed households, % | Socioeconomic features | 0.0847 |
|  | Demolitions/sq mi, N | Public goods & services | 0.0772 |
|  | Parcels tax delinquent, % | Socioeconomic features | 0.0628 |
|  | Population Black alone, % | Demographic features | 0.0578 |
|  | Population aged 16+ out of the labor force, % | Socioeconomic features | 0.0500 |
|  | Population aged 15 to 29 and male, % | Demographic features | 0.0491 |
| Homicide | Population white alone, % | Demographic features | 0.2162 |
|  | Damaged buildings/sq mi, N | Building quality & type | 0.1699 |
|  | Female-headed households, % | Socioeconomic features | 0.1232 |
|  | Population Black alone, % | Demographic features | 0.1105 |
|  | Single family homes/sq mi, N | Building quality & type | 0.1050 |
|  | Commercial buildings/sq mi, N | Building quality & type | 0.0794 |
|  | Multi-family homes/sq mi, N | Building quality & type | 0.0749 |
|  | Large apartment buildings/sq mi, N | Building quality & type | 0.0709 |
|  | Population aged 16+ unemployed, % | Socioeconomic features | 0.0693 |
|  | Median residential sales price in past 5 years | Socioeconomic features | 0.0659 |
| **DETROIT** |  |  |  |
| Firearm Homicide | Single family homes/sq mi, N | Building quality & type | 0.2421 |
|  | Population | Demographic features | 0.1209 |
|  | HOLC’s redlining grade | Historical features | 0.0976 |
|  | Population Black alone, % | Demographic features | 0.0533 |
|  | Parcels tax delinquent, % | Socioeconomic features | 0.0476 |
|  | Kids aged 3+ enrolled in nursery/preschool, % | Socioeconomic features | 0.0380 |
|  | Small apartment buildings/sq mi, N | Building quality & type | 0.0357 |
|  | Female-headed households, % | Socioeconomic features | 0.0301 |
|  | Damaged buildings/sq mi, N | Building quality & type | 0.0287 |
|  | Racial residential segregation | Demographic features | 0.0243 |
| Homicide | Single family homes/sq mi, N | Building quality & type | 0.3493 |
|  | Population | Demographic features | 0.0673 |
|  | Small apartment buildings/sq mi, N | Building quality & type | 0.0552 |
|  | Road network density | Public goods & services | 0.0530 |
|  | Female-headed households, % | Socioeconomic features | 0.0502 |
|  | Racial residential segregation | Demographic features | 0.0416 |
|  | Residential economic segregation | Socioeconomic features | 0.0370 |
|  | Large apartment buildings/sq mi, N | Building quality & type | 0.0305 |
|  | Population Black alone, % | Demographic features | 0.0282 |
|  | Population aged 25+ with at least a high school education, % | Socioeconomic features | 0.0193 |

**Appendix Table 5. Variables Included and Excluded in Sensitivity Analysis Removing Highly Correlated Variables**

| **Included Variables** | **Exclude Variables** |
| --- | --- |
| ***Building Quality & Type*** | |
| Damaged buildings/sq mi | Mixed-use parcels/sq mi |
| Plurality of parcel types |  |
| Single family homes/sq mi |  |
| Small apartment buildings/sq mi |  |
| Multi-family homes/sq mi |  |
| Large apartment buildings/sq mi |  |
| Condominiums/sq mi |  |
| Commercial buildings/sq mi |  |
| Industrial buildings/sq mi |  |
| Vacant lots/sq mi |  |
| Parcels with vacant buildings, % |  |
| ***Public Goods & Services*** | |
| Primary and secondary education schools/sq mi | Rehabilitations/sq mi |
| Hospitals/sq mi |  |
| Federally qualified health centers/sq mi |  |
| Land protected from development, % |  |
| Religious buildings/sq mi |  |
| On-premises alcohol outlets/sq mi |  |
| Off-premises alcohol outlets/sq mi |  |
| Road network density |  |
| Minimum distance to nearest transit stop |  |
| National Walkability Index |  |
| Demolitions/sq mi |  |
| ***Residential Stability*** | |
| Occupied housing units renter-occupied, % | Maximum number of arm’s length transfers for a single parcel |
| Recent movers |  |
| Parcels with arm’s length transfer in past 5 years, % |  |
| ***Socioeconomic Features*** | |
| Currently in the armed forces | Female-headed households, % |
| Residential economic segregation | Population aged 16+ unemployed, % |
| Median household income | Population aged 16+ out of the labor force, % |
| Households with crowding, N | Employed civilian population 16+ working in high-paying professions, % |
| Population using public transportation | Population aged 25+ with at least a high school education, % |
| Median residential sales price in past 5 years | Kids aged 3+ enrolled in nursery/preschool, % |
| Parcels tax delinquent, % | Kids enrolled in K-12 in private school, % |
|  | Population below 150% of poverty line, % |
|  | Parcels tax foreclosed, % |
|  | Parcels mortgage foreclosed, % |
| ***Historical Features*** | |
| HOLC’s redlining grade |  |
| ***Demographic Features*** | |
| Population | Population aged 15 to 29 and male, % |
| Racial residential segregation | Population Hispanic or Latino, % |
| Population foreign born and naturalized | Population Black alone, % |
| Population foreign born and not a citizen | Population white alone, % |
|  | Population other race or 2+ races, % |

**Appendix Table 6. XGBoost Model Performance,* Sensitivity Analysis Removing Highly Correlated Variables**

| **Outcome** | **Observed**  **Mean (SD)** | **Predicted**  **Mean (SD)** | **Pearson’s *r*** | **RMSE** |
| --- | --- | --- | --- | --- |
| CLEVELAND |  |  |  |  |
| Pt 1.  Violence | 29.27 (18.22) | 29.61 (15.77) | 0.87 | 8.92 |
| Pt. 1 Firearm  Violence | 5.44 (4.43) | 5.66 (3.80) | 0.65 | 3.47 |

* Measured using the testing data

**Appendix Table 7. Ten Most Important Predictors of Violence, Sensitivity Analysis Removing Highly Correlated Variables**

| **Outcome** | **Variable** | **Category** | **Mean Value** |
| --- | --- | --- | --- |
| CLEVELAND |  |  |  |
| Pt. 1 Violence | Multifamily (2-4 family) homes per square mile | Building quality & type | 0.1695 |
|  | Road network density | Public goods & services | 0.1174 |
|  | Racial residential segregation | Demographic features | 0.0944 |
|  | Commercial buildings per square mile | Building quality & type | 0.0824 |
|  | Percent of population that uses public transportation | Socioeconomic features | 0.0640 |
|  | Median household income | Socioeconomic features | 0.0408 |
|  | Occupied housing units renter-occupied, % | Residential stability | 0.0399 |
|  | Residential economic segregation | Socioeconomic features | 0.0366 |
|  | Damaged buildings/sq mi, N | Building quality & type | 0.0344 |
|  | National Walkability Index | Public goods & services | 0.0339 |
| Pt 1. Firearm Viol | Racial residential segregation | Demographic features | 0.2096 |
|  | Damaged buildings/sq mi, N | Building quality & type | 0.2005 |
|  | Multifamily (2-4 family) homes per square mile | Building quality & type | 0.1528 |
|  | Median residential sales price in past 5 years | Socioeconomic features | 0.0995 |
|  | Commercial buildings per square mile | Building quality & type | 0.0893 |
|  | Parcels tax delinquent, % | Socioeconomic features | 0.0658 |
|  | HOLC’s redlining grade | Historical features | 0.0593 |
|  | Residential economic segregation | Socioeconomic features | 0.0523 |
|  | Road network density | Public goods & services | 0.0427 |
|  | Single family homes/sq mi, N | Building quality & type | 0.0403 |

* X and Y coordinates were both in the top 10 Shapley values for Part 1 violence and X was in the top 10 for Part 1 firearm violence; they are not included in the table as they were included only to control for spatial autocorrelation.
